# Effect of Material Parameter of Viscoelastic Giesekus Fluids on Extensional Properties in Spinline and Draw Resonance Instability in Isothermal Melt Spinning Process

**DOI:** 10.3390/polym13010139

**Published:** 2020-12-31

**Authors:** Geunyeop Park, Jangho Yun, Changhoon Lee, Hyun Wook Jung

**Affiliations:** 1 Department of Chemical and Biological Engineering, Korea University, Seoul 02841, Korea; gypark@grtrkr.korea.ac.kr (G.P.); forza@grtrkr.korea.ac.kr (C.L.); 2 Hyundai Oilbank, Central Technology Research Institute, Yongin 16891, Korea; jjangho99yun@gmail.com

**Keywords:** viscoelastic spinning, draw resonance, kinematic waves, extensional deformation, stability indicator, Giesekus fluid

## Abstract

The draw resonance instability of viscoelastic Giesekus fluids was studied by correlating the spinline extensional features and transit times of several kinematic waves in an isothermal melt spinning process. The critical drawdown ratios were critically dependent on the Deborah number (*De*, the ratio of material relaxation time to process time) and a single material parameter ($\alpha_{G}$) of the Giesekus fluid. In the intermediate range of $\alpha_{G}$, the stability status changed distinctively with increasing *De*, i.e., the spinning system was initially stabilized and subsequently destabilized, as *De* increases. In this $\alpha_{G}$ regime, the level of velocity and extensional-thickening rheological property in the spinline became gradually enhanced at low *De* and weakened at high *De*. The draw resonance onsets for different values of $\alpha_{G}$ were determined precisely using a simple indicator composed of several kinematic waves traveling the entire spinline and period of oscillation. The change in transit times of kinematic waves for varying *De* adequately reflected the effect of $\alpha_{G}$ on the change in stability.

## 1. Introduction

**Figure 1:**
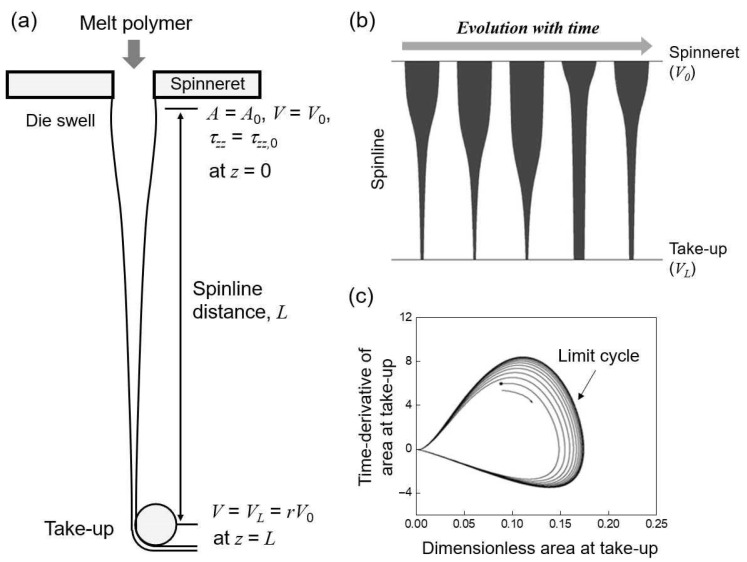
Schematics of (**a**) melt spinning process with spinline conditions, (**b**) periodic oscillation of filament during draw resonance, (**c**) limit cycle of spinline area at take-up position over the critical drawdown ratio.

Fiber spinning is one of the representative extensional deformation polymer processes, fabricating highly oriented fibers with large drawdown ratios ($r=V_{L}/V_{0}$) of velocities at the take-up ($V_{L}$) and spinneret ($V_{0}$) positions in the spinline (Figure 1a) [1]. The product quality and processability of the fibers are greatly influenced by the rheological properties of the polymeric filaments and spinline conditions. The most important concern to ensure the uniformity of the fibers is the stability of the spinning flow in the spinline from the spinneret to the take-up positions. The well-known instability in the spinning flow is draw resonance that is characterized by self-sustained periodic oscillations of state variables such as fiber diameter and spinline tension (Figure 1b), when the drawdown ratio exceeds the critical value. This was first observed by Christensen [2] and Miller [3]. Subsequently, various theoretical and experimental developments on this phenomenon were reported in melt spinning processes with various complex fluids such as Newtonian, viscous, and viscoelastic ones [4,5] using linear stability analysis [6,7,8,9,10], direct transient responses [11], kinematic traveling waves [12,13,14,15], bifurcation theory [16,17], and experimental observations [18,19].

The investigation of basic spinning flow as a uniaxial extensional deformation process has been a well-known classical topic in the last four to five decades in academia and industries. The linear stability method, focusing on the theoretical and numerical aspects of the draw resonance, is very useful for determining critical onsets in various spinning processes by transforming nonlinear governing equations into eigen-systems linearized by infinitesimal disturbances based on steady states. The nonlinear periodic oscillations of state variables beyond the onsets, exhibiting limit cycles (Figure 1c), were elucidated using direct transient simulations. Hyun and coworkers [12,13,14,15] tried to address the fundamental physics behind draw resonance by incorporating kinematic waves penetrating the entire spinline as the stability indicator. Draw resonance was found to be a type of supercritical Hopf bifurcation using the bifurcation theory [16,17]. Recently, Kwon et al. [20] determined draw resonance onsets precisely using the transfer function method under the constant force boundary condition that rendered the system constantly stable.

The exact value of the critical drawdown ratio for Newtonian fluids is known to be 20.218 for an isothermal spinning flow without any secondary forces. Based on this value, various stability windows for generalized Newtonian and viscoelastic fluids were established, depending on their material properties. The shear-thinning nature (when the power-law index, *n*, is less than one) makes the system less stable [21]. Viscoelasticity results in dichotomous behavior of the onsets with respect to the Deborah number (*De*), defined as $\lambda V_{0}/L$ (a dimensionless number representing the ratio of a material relaxation time to a characteristic time for the deformation process, where *λ* is the material relaxation time and *L* is the spinline length), stabilizing for an extensional-thickening fluid such as low-density polyethylene (LDPE) and destabilizing for an extensional-thinning fluid such as high-density polyethylene (HDPE) with *De*. For instance, White–Metzner [8,22] and Phan-Thien and Tanner (PTT) fluids [23] showed distinct dichotomous stability curves with respect to *De*, depending on each material parameter characterizing the extensional feature in their fluid models. In this study, we attempted to explain the nature of the stability curves in the spinning process of Giesekus fluids—initially stabilizing and subsequently destabilizing pattern with respect to *De* in the intermediate range of the material parameter. It is important to take into consideration the relationship between the stability window and spinline extensional characteristics for viscoelastic Giesekus fluids, which demonstrate an unusual dependence of the spinning stability on *De* at the intermediate values of material parameter. The Giesekus fluid model [24,25] is a prominent constitutive equation that reflects the realistic viscoelastic features of polymeric liquids and successfully predicts the material functions in extensional as well shear flows using a singlematerial parameter [26]. This fluid was reliably implemented to investigate polymer extensional deformation processes such as fiber spinning [27], film casting [28,29], and film blowing [30].

Various theoretical approaches were considered in this study to examine the changes in the stability curves with respect to the material parameter of Giesekus fluids in the isothermal spinning process without cooling, including steady velocity profiles and extensional deformation properties in the spinline, and kinematic waves traveling along the entire spinline.

## 2. Simulation Methods

### 2.1. Governing Equations of Spinning Flows

Simplified one-dimensional governing equations for the isothermal spinning flow of Giesekus fluids are given here under the following assumptions: (1) The equation set neglects radial stress and all secondary forces such as inertia, gravity, surface tension, and air drag. Including them will not change the fundamental aspects described here. (2) The origin at the maximum position of the die swell excludes the pre-shear history in the nozzle. (3) The fiber is slender with uniform properties in the cross-section [15,20].

Equation of continuity (EOC):

(1) $$\frac{\partial a}{\partial t}+\frac{\partial (av)}{\partial x}=0$$

where $a=\frac{A}{A_{0}}$, $v=\frac{V}{V_{0}}$, $x=\frac{z}{L}$, $t=\frac{t^{*}V_{0}}{L}$

Equation of motion (EOM):

(2) $$\frac{\partial }{\partial x}(a\tau )=0$$

where $\tau =\frac{2\tau_{zz}L}{\eta V_{0}}$

Constitutive Equation (CE, Giesekus model):

(3) $$\tau +De\left[\frac{\partial \tau }{\partial t}+v\frac{\partial \tau }{\partial x}-2\tau \frac{\partial v}{\partial x}\right]+2\alpha_{G}\tau^{2}De=\frac{\partial v}{\partial x}$$

Boundary conditions:

(4) $$a=1,\;v=1,\;\tau =\tau_{0}\;\mathrm{at}\;x=0,\;\mathrm{and}\;v=r\;\mathrm{at}\;x=1$$

The aforementioned equations are non-dimensionalized using the following dimensionless variables; a denotes the dimensionless spinline cross-sectional area of *A*, v is the dimensionless spinline velocity of *V*, *t* is the dimensionless time of t^*^, *x* is the dimensionless spatial coordinate of *z*, τ is the dimensionless axial stress of $\tau_{zz}$, *De* is the Deborah number, and r is the drawdown ratio. $\alpha_{G}$ represents a material parameter portraying the extensional behavior of the Giesekus fluid. The subscripts 0 and *L* represent the spinneret and take-up positions, respectively.

The steady velocity profiles along the spinline and corresponding apparent extensional properties were solved using the 4th-order Runge–Kutta method, ensuring the acceptable level of numerical accuracy.

### 2.2. Linear Stability Analysis of Steady Flows

The governing Equations (1)–(3) were linearized using the following perturbation variables based on steady states for constructing the linear eigen-systems.

(5) $$a(t,x)=a_{s}(x)+\alpha (x)e^{\Omega t},\;v(t,x)=v_{s}(x)+\beta (x)e^{\Omega t},\;\tau (t,x)=\tau_{s}(x)+\gamma (x)e^{\Omega t}$$

where, the subscript *s* denotes the steady state and Ω is the complex eigenvalue. *α*, *β*, and *γ* are infinitesimal perturbed quantities (i.e., eigenvectors).

Linearized EOC:

(6) $$\Omega \alpha =\left(\frac{v_{s}{}^{\prime }}{v_{s}{}^{2}}\right)\beta -\left(\frac{1}{v_{s}}\right)\beta^{\prime }-\left(v_{s}{}^{\prime }\right)\alpha -\left(v_{s}\right)\alpha^{\prime }$$

Linearized EOM:

(7) $$0=-\left(\frac{v_{s}{}^{\prime }}{v_{s}{}^{2}}\right)\gamma +\left(\frac{1}{v_{s}}\right)\gamma^{\prime }+\left(\tau_{s}{}^{\prime }\right)\alpha +\left(\tau_{s}\right)\alpha^{\prime }$$

Linearized CE:

(8) $$\Omega \gamma =-\tau_{s}{}^{\prime }\beta +\left(2\tau_{s}+\frac{1}{De}\right)\beta^{\prime }+\left(2v_{s}{}^{\prime }-\frac{1}{De}-4\alpha_{G}\tau_{s}\right)\gamma -v_{s}\gamma^{\prime }$$

Boundary conditions:

(9) α(0) = β(0) = 0 at x= 0 and β(1)=0 at x = 1

The boundary conditions (given by Equation (9)) indicate that the flow rate at the spinneret and velocity at the take-up position are unperturbed under constant velocity operation. The prime (′) symbol signifies the derivative with respect to *x*. The critical drawdown ratios, when the real part of the first normal mode is zero, are obtained by solving the eigenvalues from the linear eigen-system, $\Omega \underline{\underline{M}}\underline{y}=\underline{\underline{A}}\underline{y}$, where $\underline{y}=\left[\underline{\alpha },\;\underline{\beta },\;\underline{\gamma }\right]$ via the shift-invert method [15]. They are plotted with respect to *De* for different values of $\alpha_{G}$.

### 2.3. Simple Stability Indicator Using Traveling Times of Kinematic Waves

Draw resonance is a hydrodynamic instability that can be physically figured out by several kinematic waves penetrating the entire spinline from the spinneret to the take-up position [13,14]. The stability criterion (Equation (10)) comprising the unity-throughput wave, maximum/minimum area wave, and period of oscillation was confirmed to correctly interpret the draw resonance dynamics for various fluid systems [4,5].

(10) $${\left(t_{L}\right)}_{1}+{\left(t_{L}\right)}_{2}+\frac{T}{2}\;\;\begin{array}{c} >\\ =\\ < \end{array}\;\;{\left(\theta_{L}\right)}_{1}+{\left(\theta_{L}\right)}_{2}\;\mathrm{for}\;r\begin{array}{c} >\\ =\\ < \end{array}r_{c}$$

where ${\left(t_{L}\right)}_{1}$ and ${\left(t_{L}\right)}_{2}$ represent the dimensionless traveling times of the unity-throughput waves, ${\left(\theta_{L}\right)}_{1}$ and ${\left(\theta_{L}\right)}_{2}$ are the dimensionless traveling times of the maximum and minimum cross-sectional area waves, and T is the dimensionless period of oscillation.

In the case of $r<r_{c}$, the left-hand side (LHS; designated as the required time) of Equation (10) becomes larger than the right-hand side (RHS; designated as the allowed time), implying that the oscillation cannot be sustained due to insufficient time for inducing the draw resonance [12,13]. For $r=r_{c}$, both sides of Equation (10) are identical, triggering the draw resonance. When $r>r_{c}$, the magnitude of both sides is reversed and periodic oscillation by draw resonance is continuously maintained. Although the real traveling times and period data must be acquired by the direct transient simulation of nonlinear governing equations, it was shown that the simplified version of the stability indicator from the linear stability analysis could reliably determine the onsets [15]. In this study, we tried to identify the size change on both sides of Equation (10) using linear eigen-mode data.

## 3. Results and Discussion

### 3.1. Neutral Stability Curves with Respect to De for Giesekus Fluids with Different $\alpha_{G}$ Values

**Figure 2:**
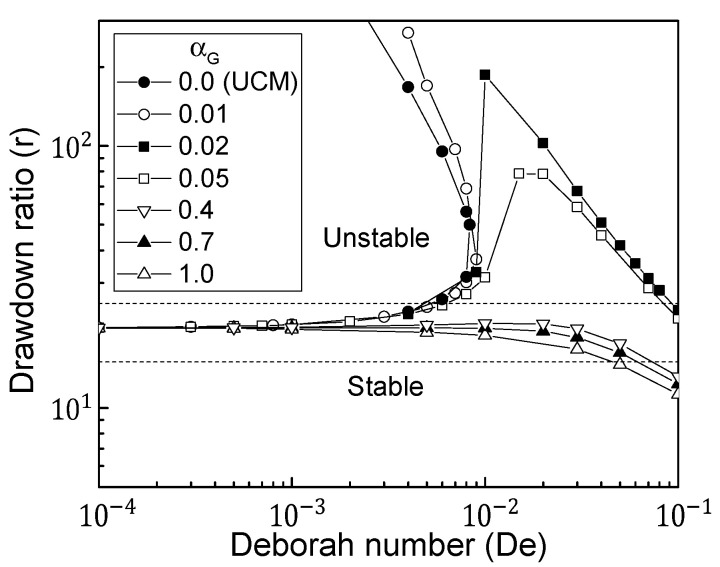
Stability windows of Giesekus fluids for various $\alpha_{G}$ values. Here, $\alpha_{G}$ represents a material parameter of the Giesekus fluid. If $\alpha_{G}=0,$ it is identical to the upper-convected Maxwell (UCM) model.

As illustrated in Figure 2, the critical drawdown ratios for Giesekus fluids were determined from the first normal mode of the linearized spinning systems, when *De* and $\alpha_{G}$ were varied. The extensional properties (extensional-thickening or -thinning) of fluids played a significant role in the stability of various spinning processes. Unlike other viscoelastic models such as White–Metzner and PTT fluids [8,31], the Giesekus fluid exhibited interesting stability patterns depending on the value of $\alpha_{G}$. Three stability patterns can be shown in Figure 2. When $\alpha_{G}$ was less than about 0.01, *De* rendered the system more stable implying that the critical drawdown ratio increased with increasing *De* and a secondary stable region at high draw ratios was observed, as in the typical extensional-thickening case (e.g., LDPE) of an upper-convected Maxwell (UCM) fluid with $\alpha_{G}$= 0. It is noted that the PTT fluid under extensional-thickening conditions did not show the secondary stable region [31].

When $\alpha_{G}$ was in the intermediate range 0.01–0.4, the system was first stabilized and then destabilized as *De* increased, distinguishing it from other viscoelastic fluids. The extensional deformation features in the spinline, as described in the next section, qualitatively explain the trend in the stability of the Giesekus fluid for intermediate value of $\alpha_{G}$. A value of $\alpha_{G}$ greater than about 0.4 made the system less stable to disturbances with increasing *De*; this was frequently observed in extensional-thinning fluids such as HDPE.

### 3.2. Steady Extensional Properties of Giesekus Fluids in the Spinline

**Figure 3:**
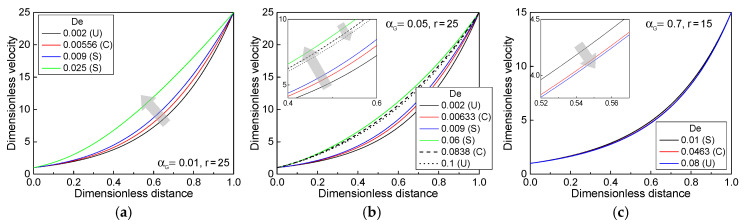
Dimensionless steady spinline velocity profiles under several material parameter conditions: (**a**) $\alpha_{G}$ = 0.01, *r* = 25, (**b**) $\alpha_{G}$ = 0.05, *r* = 25, and (**c**) $\alpha_{G}$ = 0.7, *r* = 15. S, C, and U in the box indicate stable, critical, and unstable states, respectively. The arrow indicates the direction in which *De* increases.

The aforementioned neutral stability curves for various values of $\alpha_{G}$ are basically associated with the extensional behavior and properties in the spinline. First, the steady velocity profiles around the onsets for varying *De* in three fluid cases ((a) $\alpha_{G}$ = 0.01, *r* = 25; (b) $\alpha_{G}$ = 0.05, *r* = 25; and (c) $\alpha_{G}$ = 0.7, *r* = 15) were compared and are shown in Figure 3. The extensional-thickening fluid with $\alpha_{G}$ = 0.01 showed a higher velocity level in the same spinline position, as *De* increased (Figure 3a), requiring less residence time along the spinline with increasing *De*. However, the spinline velocity profiles in Figure 3c for the case of the extensional-thinning fluid with $\alpha_{G}$ = 0.7 were the opposite with respect to the previous case, i.e., gradually decreasing velocity level in the same spinline position with increasing *De*. For intermediate value of $\alpha_{G}$, the dependence of the steady velocity profiles on *De* was different around the first and second drawdown ratio onsets at low and high *De* regions, exhibiting a higher spinline velocity level near the first onset and subsequent lower velocity level around the second onset, as *De* increased (Figure 3b).

**Figure 4:**
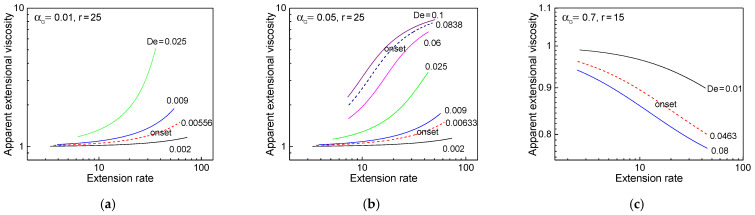
Apparent extensional viscosity in the spinline with respect to extension rate under several material parameter conditions: (**a**) $\alpha_{G}$ = 0.01, *r* = 25, (**b**) $\alpha_{G}$ = 0.05, *r* = 25, and (**c**) $\alpha_{G}$ = 0.7, *r* = 15.

The extension rate ($\dot{\varepsilon }$) and apparent extensional viscosity ($\eta_{E}$) were evaluated from the steady velocity and tensile stress profiles in the spinline, respectively, under the spinning conditions shown in Figure 3. The apparent extensional viscosity rapidly increased with increasing *De* for the extensional-thickening case with $\alpha_{G}$ = 0.01, as shown in Figure 4a. It must be noted that the non-zero value of $\alpha_{G}$ in this case effectively prevents the infinite growth of tensile stress [32]. In the case of intermediate value of $\alpha_{G}$ (Figure 4b), the growth rate of the extensional viscosity with increasing *De* is analogous to the case of $\alpha_{G}$ = 0.01 around the lower onset, but not significant around the higher onset, as compared to that shown in Figure 4a. The extensional-thinning fluid (Figure 4c) showed decreasing extensional viscosity with respect to the extension rate and lower viscosity level with increasing *De*, as expected.

### 3.3. Transit Times of Kinematic Waves for Different Giesekus Fluids

**Figure 5:**
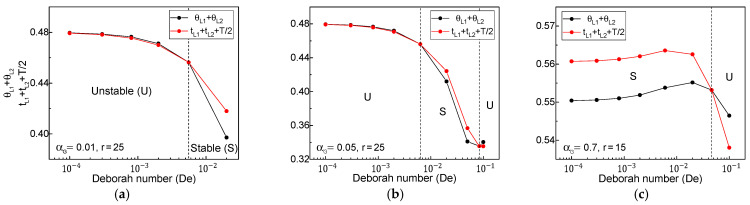
Changes in time scales of left-hand side (LHS) and right-hand side (RHS) of the simple indicator: (**a**) $\alpha_{G}$ = 0.01, *r* = 25, (**b**) $\alpha_{G}$ = 0.05, *r* = 25, and (**c**) $\alpha_{G}$ = 0.7, *r* = 15. Here, $\theta_{L1}$ and $\theta_{L2}$ represent the dimensionless traveling times of the maximum and minimum cross-sectional area waves; $t_{L1}$ and $t_{L2}$ are the dimensionless traveling times of the unity-throughput waves; T is the dimensionless period of oscillation.

Figure 5 displays the changes in the LHS and RHS times (composed of the traveling times of two kinematics waves and period of oscillation) of the simple stability indicator (Equation (10)) with respect to *De*, to confirm the draw resonance onsets for three Giesekus fluids with $\alpha_{G}$ = 0.01, 0.05, and 0.7. As *De* increased, the magnitude of the LHS and RHS times crossed exactly at critical points. For instance, the extensional-thickening case for $\alpha_{G}$ = 0.01 was stable after a critical *De* = 0.00556 at *r* = 25; the intermediate case for $\alpha_{G}$ = 0.05 was stable only in the range of *De* = 0.00633–0.0838 at *r* = 25; the extensional-thinning case for $\alpha_{G}$ = 0.7 became unstable after a critical *De* = 0.0463 at *r* = 15. It was observed that these onsets were identical to those shown in Figure 1 obtained from the linear stability analysis.

**Figure 6:**
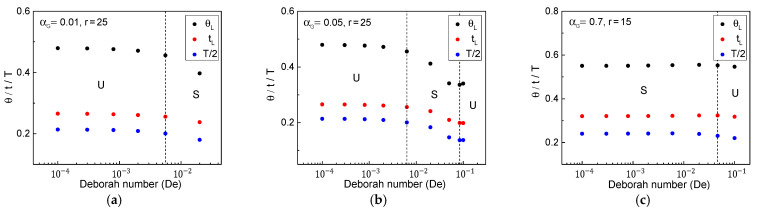
Traveling times of unity-throughput wave ($t_{L}$) and maximum/minimum cross-sectional area wave ($\theta_{L}$), and period of oscillation (*T*) for (**a**) $\alpha_{G}$ = 0.01, *r* = 25, (**b**) $\alpha_{G}$ = 0.05, *r* = 25, and (**c**) $\alpha_{G}$ = 0.7, *r* = 15.

Figure 6 compares each traveling time of the kinematic waves and period of oscillation with respect to *De* for the three fluid cases illustrated in Figure 5. Interestingly, the traveling time of the maximum or minimum cross-sectional area wave showed a slight upward turn after the second critical point at a higher drawdown ratio, as shown in Figure 6b, as qualitatively described in the steady velocity profiles.

## 4. Conclusions

Neutral stability curves for Giesekus fluids were established in the melt spinning processes. The material parameter $\alpha_{G}$ in this fluid model suitably depicted the extensional-thickening (stabilizing effect of *De*) and extensional-thinning (destabilizing effect of *De*) properties of viscoelastic fluids in extensional deformation processes. In the intermediate range of values of $\alpha_{G}$ (approximately 0.01 < $\alpha_{G}$ < 0.4), the effect of *De* on the stability was unusual—the system was stabilized in the low and medium *De* regions and then destabilized in the high *De* region. This tendency may be qualitatively interpreted by extensional flow characteristics in the spinline. When *De* increased at a fixed drawdown ratio (e.g., *r* = 25 condition applied in this study) in the intermediate $\alpha_{G}$ region, the system, starting from the unstable state, became stable after the first onset for low or medium *De*, resulting in a higher level of spinline velocity and strain-hardening viscosity. It became unstable again beyond the second onset for high *De*, yielding a lowered spinline velocity and an insignificant strain-hardening feature. A combination of transit times of the kinematic waves penetrating the entire spinline and period of oscillation, i.e., the simple indicator from the linear stability analysis, predicted well the draw resonance onsets under different *De* and $\alpha_{G}$ conditions. It was confirmed that these transit times of kinematic waves for varying *De* adequately reflected the dependence of change in stability on the values of $\alpha_{G}$.

## Data Availability

The data presented in this study are available on request from the corresponding author.
